# Making ‘safer injecting’ matter for people who inject image and performance enhancing drugs

**DOI:** 10.1177/13634593251388294

**Published:** 2025-11-08

**Authors:** Timothy Piatkowski, Emma Kill, Sonya Weith, Steph Reeve, Luke Cox, Ross Coomber, Cheneal Puljevic, Thomas O’Connor, Jason Ferris

**Affiliations:** 1 Griffith University, Gold Coast, QLD, Australia; 2 The University of Queensland, Brisbane, Australia; 3 Queensland Injectors Voice for Advocacy and Action, Sunshine Coast, Australia; 4 Swansea University, Wales, UK; 5 University of Liverpool, UK; 6 University of Connecticut School of Medicine, Farmington, USA

**Keywords:** anabolic-androgenic steroids, blood-borne virus, image and performance enhancing drugs, infection, injecting

## Abstract

Image and performance-enhancing drugs (IPEDs) are primarily injected intramuscularly or subcutaneously, yet traditional harm reduction strategies, focused on blood-borne virus prevention, often overlook the specific health enhancement goals of people who use IPEDs. This study advocates for a collaborative approach to safer injecting practices, informed by the lived-living experiences of people injecting IPEDs, to develop targeted messaging that aligns with their unique motivations. Thirty participants who inject IPEDs were recruited through community networks and partnerships. The study used qualitative focus groups and semi-structured interviews to explore injection practices and safety strategies. Data analysis followed an inductive, line-by-line approach, identifying themes related to injection methods and safer techniques, drawing on matters-of-concern. Stigma and misinformation about IPED injection practices were prevalent, contributing to a heightened risk of infection. Participants expressed anxiety about injection site reactions and reported limited knowledge of safe techniques, with poor injecting literacy leading to risky behaviours like needle reuse and inadequate hygiene. Importantly, participants highlighted that IPED injecting knowledge is often shared through peer support networks. This research calls for reframing harm reduction to focus on health enhancement, optimising injecting techniques, and integrating evidence-based resources, empowering peer-led harm reduction to better support the health aspirations of this population.

## Introduction

Image and performance-enhancing drugs (IPEDs), such as anabolic-androgenic steroids (AAS), are consumed via multiple routes including oral, topical, and injection (McVeigh et al., 2022). Oral use of anabolic-androgenic steroids (AAS) is common among consumers, with around one third consuming these drugs orally (van de Ven et al., 2020). However, injection remains the predominant route for many IPED substances, with approximately 70% of consumers injecting in the UK (Hope et al., 2015). AAS are typically administered intramuscularly using larger gauge needles (e.g., 23G), targeting major muscle groups such as the gluteus maximus (i.e., buttocks) or vastus lateralis (i.e., upper thigh) (Hope et al., 2015). Other IPEDs, including human growth hormone (HGH) and off-label medicines like insulin, are usually injected subcutaneously (Piatkowski and Cox, 2024). These routes of administration and substances differ significantly from those associated with other forms of drug injection, which often involve intravenous use and smaller gauge needles (e.g., 31G). These technical differences reflect deeper divergences in risk environments (Santos and Coomber, 2017), motivations (Kimergård and McVeigh, 2014), and social identities (Piatkowski et al., 2023). This body of literature has extensively documented the diverse practices, motivations, and risk perceptions that characterise IPED consumers, providing important insights into their health-related decision-making processes (Andreasson and Henning, 2022; McVeigh et al., 2016; Santos and Coomber, 2017; Underwood, 2017).

People who inject IPEDs often do so within highly structured and deliberate routines aimed at achieving specific goals related to body image, performance, and health. Their injecting practices, risk perceptions, and substance choices differ substantially from those of people who inject psychoactive drugs (Fomiatti et al., 2019, 2020; Fraser et al., 2020; Keane, 2005; Kimergård and McVeigh, 2014; Latham et al., 2019; Santos and Coomber, 2017). There is a substantial evidence base exploring these factors, including the social, psychological, and cultural drivers behind IPED use (Brennan et al., 2017; McVeigh et al., 2021), although nuanced aspects of how these motivations translate into injecting practices and harm reduction behaviours remain under-examined. While there are documented instances of HIV and other blood-borne viruses (BBVs) among this population (Hope et al., 2015, 2021), these are not typically the central concerns of people who use IPEDs. Rather, issues such as contamination, improper injecting technique, and complications from non-medical dosages are more frequently cited as meaningful health risks (Grant et al., 2023; Havnes et al., 2019; Hope et al., 2021; Zahnow et al., 2017).

Despite these distinct priorities, harm reduction responses for IPED consumers have largely been adapted from models originally developed for people who inject psychoactive drugs. This has resulted in interventions, such as needle and syringe programs (NSPs), that often fall short of meeting the nuanced needs of people who use IPEDs (Bates et al., 2021; Graf et al., 2022; Kimergård, 2015; McVeigh et al., 2021; van de Ven et al., 2018). For instance, NSPs may not routinely stock the appropriate injecting equipment (e.g., larger-gauge needles or longer barrels for intramuscular use), nor do they always provide tailored advice on safer injection techniques specific to IPED substances (Piatkowski et al., 2022). Staff may lack training in IPED pharmacology or be unaware of the distinct health and aesthetic goals that motivate use, limiting their capacity to provide relevant health information (Piatkowski et al., 2024a). Moreover, services are sometimes perceived as stigmatising or irrelevant by IPED consumers (Cox et al., 2024; McVeigh et al., 2022; Piatkowski et al., 2022), especially when the primary focus remains on BBV prevention (Palmateer et al., 2010, 2022; Treloar et al., 2016). This narrow focus does not adequately address the broader spectrum of risks associated with IPED use, such as contamination, abscesses, endocrine disruption, or mental health concerns (Craven et al., 2025; Piatkowski et al., 2025a, 2025b). Scholars have therefore questioned whether BBV prevention should remain the central organising principle for this population’s care (Bates et al., 2021; Underwood, 2019).

More recently, there has been increasing consensus that harm reduction approaches for IPED consumers need to expand beyond traditional BBV prevention to encompass the full range of health and wellbeing concerns relevant to this population (Bates et al., 2021; Jacka et al., 2020; Rowe et al., 2017; Underwood, 2019). However, translating these policy and theoretical shifts into consistent, accessible services remains challenging, particularly in non-urban areas (Piatkowski et al., 2024a; Turnock and Mulrooney, 2023). To address these gaps, harm reduction must evolve through innovative, peer-informed approaches. Involving people with lived-living experience in the design and delivery of services enhances relevance, fosters trust, and ensures that strategies reflect the realities of those who use IPEDs. To address these gaps, this study aims to explore the lived-living experiences of people who inject IPEDs, focusing on their injecting practices and understandings of safer injecting.

### Approach

We draw on the work of Science and Technology Studies scholars John Law (2002) and Annemarie Mol (2002), particularly the ontological turn. Their work invites us to rethink foundational assumptions in health and drug research by viewing knowledge, care, and material practices not as stable or universal, but as multiple, situated, and enacted (Law, 2002; Mol, 2002). This framing provides a valuable lens for examining how safer injecting practices for people who inject IPEDs are not merely adopted or resisted, but actively co-produced through social, technical, and institutional arrangements. Specifically, we draw on Law’s (2002) notion of mess, which challenges the idea that research findings must be neat, coherent, and easily classified, instead embracing the complexity, uncertainty, and fluidity inherent in lived experience. We also draw on Mol’s (2002) concept of the body multiple, which suggests that the body is not a singular, stable entity but is enacted differently across social, medical, and experiential contexts. Together, these concepts help us conceptualise safer injecting not as a fixed or universal practice, but as something emergent, contingent, and multiple, shaped by context, embodiment, and relationships. Instead, it is continually enacted in different ways across clinical, policy, and peer settings. This approach encourages attentiveness to how injecting practices are shaped through particular routines, materials (e.g., injecting equipment), spaces (e.g., clinics, gyms), and embodied experiences. These enactments have *ontological effects*: they help constitute the realities of what counts as ‘safer’, ‘harmful’, or ‘expert’ in ways that matter deeply for people who use IPEDs.

While Law and Mol offer a toolkit for tracing these enactments, their work is less explicitly focused on how political priorities and institutional power shape which enactments are recognised and legitimised. To address this, we integrate Bruno Latour’s (2004) concept of *matters of concern*, which shifts attention away from ‘debunking’ knowledge claims and toward understanding how specific facts, practices, and interventions come to matter through social, material, and political processes. Latour’s work complements Law and Mol by prompting us to ask: what concerns are mobilised in the making of safer injecting knowledge? Whose realities are included, and whose are marginalised? How are certain harm reduction strategies stabilised while others are dismissed? This addition is particularly important in our context, where the needs of people who inject IPEDs have often been overlooked within harm reduction models developed primarily for people who inject psychoactive drugs. By using *matters of concern* as an analytic, we highlight how *safer injecting* is not a neutral concept, rather it is assembled through specific configurations of care, evidence, and authority, often reflecting the concerns of policymakers or service providers rather than those of people who use IPEDs.

Taken together, these theoretical frameworks enable us to examine safer injecting not as a fixed set of behaviours, but as a *co-constituted practice* which is shaped through technical standards, regulatory constraints, and lived-living experiences. This allows us to critically reflect on how harm reduction services and knowledge practices are configured, whose concerns they prioritise, and what alternative configurations might be possible. In doing so, we aim to re-centre the realities of people who inject IPEDs, foregrounding their concerns, practices, and forms of care in efforts to reimagine safer injecting interventions.

## Methods

### Design

This research represents an exploratory and collaborative study focusing on people who inject IPEDs. Ethical approval was granted from the Griffith Universpity Human Research Ethics Committee (Approval: 2023/784).

### Sampling and recruitment

The lead author is a peer-researcher who specialises in people who use IPEDs. Participants were sourced from the lead authors well-established networks as well as through community partnerships, which included Queensland Injectors Voice for Advocacy and Action (QuIVAA). The lead author shared study details through word-of-mouth in what resembles a purposeful approach. Recruitment was voluntary and included those who expressed interest through direct contact, social media promotion, word of mouth. Once participants had been identified and interviews had been conducted, the lead author requested that the participants share the study details with their peers, known as snowballing. Informed consent was obtained verbally before each interview and was achieved through the interviewer providing a thorough overview of the study, its aims, and objectives. It was only when all the study details were known to the participants that they were able to provide informed consent. Participants were located in Australia. People who use IPEDs in Australia often remain underserved by mainstream health and alcohol and other drug services (Piatkowski et al., 2022, 2024a). All participants were currently using IPEDs and had completed one or more cycles involving multiple compounds. Substances reported included AAS such as testosterone, trenbolone, nandrolone, stanozolol, drostanolone, and oxandrolone, as well as human growth hormone and insulin. All participants obtained these substances through unregulated means. Thirty people who inject IPEDs (22 men and 8 women; median age = 32 years) participated in 25 semi-structured interviews and 1 focus group of 5 people (median length = 50 minutes; focus group length = 75 minutes).

### Data generation

The interviews were conducted between June and September 2024 by two members of the research team TP and SR via videoconferencing, were recorded and automatically transcribed, then checked and corrected, for analysis. The lead author, in collaboration with the IPED community, designed the interview questions to elicit participants’ thoughts and experiences regarding their usage. Incorporating lived-living experience into interview design ensures questions are grounded in participants’ realities, fostering cultural sensitivity and avoiding stigmatising assumptions. This approach builds trust, encourages richer responses, and empowers participants by recognising them as experts in their own lives. Specifically, prior to the interviews, the lead author engaged with members of the IPED community to discuss research priorities and areas of contention for people who inject IPEDs. This process was conducted through informal but sustained collaboration with the Steroid Advisory Group, a community reference group convened by QuIVAA. The group includes people with lived-living experience of IPED use and draws on these peer networks to inform harm reduction and research initiatives. Two members of the group undertook iterative review of the interview schedule, contributing suggestions and raising concerns to ensure it reflected the realities and nuances of IPED use. These reviews took the form of direct discussions, which were then workshopped collaboratively with the lead author. The collaborative review of questions was further underpinned by the lead authors’ living-lived experience, which provided valuable insight, garnered knowledge and demonstrated comprehensive cultural awareness of IPED use and associated injecting practices within the community.

A significant part of the discussions centred on identifying the practices required to improve health and reduce harm – and participants focused on injecting practices as part of this, demonstrating responsiveness to community care. Participants were asked about their injecting experiences with questions such as: ‘What have been your experiences with injecting?’ and ‘Have you experienced any infections or injuries from injecting?’ They were also asked about their access to healthcare, specifically, ‘How were your experiences accessing medical treatment for these?’ To explore social and structural factors influencing injecting practices, participants were prompted with: ‘How do access to healthcare services and social stigma around AAS use affect your ability to practice safer use?’ The interview also explored perceptions of available information by asking, ‘Do you think there is enough safe injecting information available?’ Finally, participants were invited to reflect on distinctions within the injecting community with the question, ‘As compared to other people who inject drugs, why are people who inject steroids different and how does this change the type of information they need?’ These questions aimed to capture both the practical challenges and the broader social context that shape safer injecting among people who use IPEDs.

### Data analysis

The lead author followed a systematic, inductive line-by-line analysis using iterative categorisation (Neale, 2016) to refine and code the data. This approach identified and developed codes related to safer use and injecting practices. Inductive thematic sufficiency was reached, and recurring themes were then organised into higher-order concepts, forming a structured framework for presenting the findings. Drawing on matters-of-concern, he began testing these concepts against the findings, using them as frameworks to interpret and integrate the data meaningfully. Importantly, the lead author’s lived-living experience of IPED injecting was consciously integrated during the abductive conceptualisation and interpretive phase (Neale, 2021). This positionality was recognised as a strength, offering an autoethnographic lens that deepened the analysis by bringing insider insight and cultural awareness inaccessible to researchers without lived-living experience (Piatkowski et al., 2025c; Reeve et al., 2025). However, this embeddedness also required rigorous reflexivity to critically examine how personal history and perspectives might shape coding, theme development, and theoretical framing (Wakeman, 2021). Wakeman (2021) highlights the value and complexity of this insider positionality, emphasising the need to balance authenticity with critical reflection. Consequently, these emotional and biographical intersections significantly shaped theoretical understandings and informed the research methodology (Wakeman, 2014). Throughout the process, the research team collectively challenged underlying assumptions and surfaced points of contention, drawing on diverse perspectives both with and without lived-living experience. When interpretive bottlenecks or divergent readings emerged, such as differing views on how to frame injecting risk or discuss importance of certain strategies, these were discussed in team meetings, allowing for reflexive dialogue that deepened the analysis and ensured conceptual clarity. The key findings are presented below, organised into three overarching theme-categories.

## Findings

### The ‘taboo’ of injection and infection

Given that a primary route of administration for IPEDs is through injection, it follows that this group will always have some level of risk to infection. However, the involvement of needles brings alongside its other connotations related to ‘injecting’:

Cherry [45, female]: As soon as you say that it’s an injectable [. . .] it involves needles. . .Needles are dirty and like you’re going to get a blood-borne illness.

These symbolic connotations become part of how injection itself is assembled as socio-affective (Piatkowski et al, 2024b) practice, where risks and meanings coalesce through material and discursive procedures. As a result, it was unsurprising that participants mentioned there is ongoing cultural stigma surrounding needles:

Cherry [45, female]: I mean, I think they’re still taboo, with anything injectable there’s taboo. If you give somebody the choice between taking Primobolan [methenolone], which is injectable and taking Anavar [oxandrolone] which is not, I reckon they’re gonna choose the Anavar in 99% of cases even if you told them that the risks [that] came with the injectable were less. Because it’s an injectable, people would tend towards taking the oral.

Gavin suggests the uncertainty many people face when considering injecting for the first time, particularly in the context of contradictory information and social stigma surrounding injection. *Gavin [31, male]: If you’ve never stuck a needle in your muscle before, it’s kind of like, oh shit, am I doing it right?.* Some participants shared personal experiences of significant injection site reactions, underscoring their concerns about potential infections that might ensue after the initial administration of IPEDs has taken place.

Damon [27, male]: My shoulder and arms were swelling up to double the size. . .Super red, like they’re gonna explode. . .is this an infection?

This fear stemmed from the inability to verify the safety of the substances they were injecting, which could be contaminated. This might occur due to substandard manufacturing procedures and the potential of fake and counterfeit products.

Damon [27, male]: I can only put down to like bacteria or something in the oil. Yeah, it was always site of injection about three to four days later, which is super weird timings as well because normally get a cork or something is there the next day or two days or whatever. This was like a delayed response, massive inflammation response to the injection site and then it was actually spreading per se.

This unexpected complication occurred despite years of experience, underscoring that even more experienced people who use IPEDs are vulnerable to contaminants and adverse reactions. These accounts demonstrate how ‘safer injecting’ becomes not just a fixed practice but an emergent concern that is continually negotiated and reassembled by consumers themselves, supporting Law’s (2002) view of practice as ontologically productive.

It is crucial to reframe the discussion around the dangers associated with consumption to reflect the realities faced by this community, recognising that infections can arise not only from unsafe injecting practices but also from contaminated products. In this sense, infection risk becomes a shifting ‘matter of concern’ (Latour, 2004), contingent on site reactions, oil composition, hygiene practices, and counterfeit supply chains, all of which are actively assembled in everyday practice.

This might strike people who IPEDs during any point within their IPED journey, underscoring the need for broad service design and delivery, catering for the needs of people across a spectrum of (un)experience.

Ross spoke to the ongoing anxiety within the community about infections and inflammatory responses at injection sites:

Ross [32. Male]: It’s a big fear for people. . . injection sites getting inflamed or infection. You can get build-up of carrier oils and hard lumps under the skin because of carrier oils.

This fear is paired with the understanding that poor hygiene practices exacerbate these risks. The description of carrier oils building up in muscles also illustrates the lesser-known risks people who inject AAS face, not just from the drugs themselves but from how they are administered. Jasmine stressed the alarming rates of infections among people who inject AAS and how many people are unconcerned about the dangers of bacterial contamination.

Jasmine [44, female]: That’s the biggest reason the steroid user is going to end up in hospital. . . with an infection. . . every time I put out a post about infections, I get flooded with photos of guys [. . .] shoulders cut open [. . .] necrotic tissue.

Here, infection is not just a biomedical outcome but a collection of concerns about purity, technique, production, and care. By treating infection this way, rather than a fixed endpoint, we can better attend to how risk and care are co-constituted through consumer experience and knowledge-making practices. This directs us to consider there may be a perception among the community underestimating the risk of bacterial infections, focusing instead on other concerns:

Raymond [42, male]: You can only have a certain amount of mls [millilitres of oil] you can put in [the muscle] and not a lot of people get told these things.

Many people prioritised concerns such as the type of oil used in injectable AAS preparations, while overlooking other commonly discussed risks like improper injection techniques, contamination, and bacterial infections. These knowledge gaps and selective priorities highlight how evidence about injecting safety is not passively received but actively filtered through local logics, experiences, and affective priorities. This focus diverts attention away from foundational aspects of safe injection practice, such as sterile techniques, needle hygiene, and site selection, which are essential for minimising infection risks.

### Contextualising risky practices for IPED injecting

Participants discussed their experiences of peers reusing needles as a result of following unverified advice from informal sources. We outline the distinction here, between needle sharing and reusing, whereby people would use the same needle on multiple occasions without disposing it. These are different problems which require and demand different harm reduction approaches. Alder reflected on the frequency of poor injecting habits, highlighting that some people may reuse needles without understanding the significant risks: *Alder [40, male]: I’ve met some guys who are just like, ‘Oh, I can just reuse the same pin’.* Reusing needles greatly increases the risk of infection (Larance et al., 2008), even if people believe they are not at risk because they are injecting IPEDs. This observation resonates with the lead author’s personal experience as someone who has injected IPEDs and AAS and, at times, reused needles. However, this behaviour was not due to misinformation, but rather a combination of convenience, accessibility issues, and the necessity to maintain hormonal balance. Sometimes, when faced with the need for frequent injections, particularly to regulate hormones, convenience can outweigh caution, leading to riskier practices. These behaviours exist along a spectrum, shaped by access, urgency, bodily needs, and competing forms of knowledge, rather than as fixed categories of safe or unsafe. Framed in this light, this helps us see how ‘risk’ is not a fixed attribute, but something assembled in situ, emerging through the entanglement of bodies, routines, materials, and informational flows (Mol, 2002).

However, many people do lack reliable guidance on how to inject IPEDs safely and proceed to do so irrespective of that literacy gap. Lena gives an example of how knowledge within the community is sometimes passed on haphazardly:

Lena [44, female]: I know so many blokes that just get injected by their girlfriend who’s never Googled where they’re supposed to inject at all like no idea. No one looks it up online, they don’t. They just go my coach’s, dog’s, cousin’s, boyfriend’s uncle showed me what to do.

Despite the availability of guidance in digital spaces and at sharps collection sites, many individuals still opt for alternative, often riskier practices where information and advice are openly available. As April explains: *April [44, female]: It is there [online], but I don’t think that they look.* This reflects how informational infrastructures are shaped not just by access but by trust, habit, and relational dynamics (Piatkowski et al., 2024c). Simply providing information about safe injecting practices, whether online or in physical spaces, is not enough to ensure it is used and implemented effectively in practice. In this sense, harm reduction advice competes with other concerns, including peer influence, urgency, or perceived expertise, highlighting how knowledge becomes actionable only when embedded in socially meaningful contexts.

The challenge goes beyond mere availability of resources; it lies in understanding how to make this information resonate with the community and motivate health enhancing practices. Current outreach efforts are failing to reach their potential. Eben emphasised that it is not enough to know what substances are being used; people must also understand how to safely inject them.

Eben [37, male]: That injecting side of the harm minimisation information is equally as important as knowing what you’re taking.

While a substantial body of research over the past two decades has sought to understand the motivations, decision-making processes, and communication preferences of people who use IPEDs (e.g., Ainsworth et al., 2022), important gaps remain. We argue that uptake of harm reduction advice should not be reduced to a simple knowledge deficit. Instead, an ontological lens that considers the misalignment between current service infrastructures and the heterogeneous practices and concerns of IPED consumers offers a more useful framework. Existing educational efforts often fail to engage with the diversity of consumers’ identities and contexts, limiting their effectiveness and uptake (e.g., McVeigh et al., 2016, 2021). Addressing these deeper social and contextual dynamics is therefore critical to developing more responsive and impactful interventions.

### ‘Safer use’ practice as safer injecting

Building trust is essential for effective engagement within communities of people who use IPEDs (Havnes and Skogheim, 2019). Participants in the current study underscored the importance of creating knowledge and messaging perceived as credible and accessible. As Anwir stated:

Anwir [37, male]: You need to be trusted. And if you’re referring them there [to the NSP] for good information anyway, that helps to have it [steroid information] there.

Trustworthy resources are pivotal for overcoming misinformation and ensuring meaningful engagement with safe injecting information. When people trust the source of information, they are more likely to seek it out and apply it in their practices. This emphasis on trust points to how knowledge infrastructures, especially those built on peer relationships, become central to whether something is ‘taken up’. Indeed, this draws upon and underscores the importance of capital (see Wacquant, 1995) within the community, with respected and known individuals capable of harnessing and influencing perceptions and the subsequent behaviours of others. Some participants suggested demonstrable and graphic imagery to give people an idea of the reality of the consequences of not implementing safe injecting techniques to have potential beneficial outcomes within harm reduction approaches.

Ross [32, male]: I think you need to [show them] what’s the worst-case scenario for them, might show them. . .infection pictures.

Lena [44, female]: Everyone loves the good, abscessed picture.

As April noted, the ‘shock factor’ not only educates people but also prompts them to share this crucial information with others: *April [44, female]: The shock factor will have people talking about it and sharing it, which is important in itself.* While the suggestion to use shock tactics, like graphic imagery, may arise naturally in peer consultations, it is important to acknowledge the limitations of this approach. Scare tactics have historically been used in public health campaigns, particularly in anti-drug initiatives, with the belief that frightening imagery deters risky behaviour (Esrick et al., 2019). However, research shows that while such tactics can grab attention, they rarely lead to long-term behaviour change and can have unintended negative effects (Hastings et al., 2004). These narratives are sometimes misinformed and can exacerbate harms associated with IPEDs, deter treatment seeking and drive perceptions of stigma within and surrounding the community. Instead, this presents an opportunity to guide the conversation toward a more progressive, evidence-based approach focused on communal knowledge exchange. Such practices, when grounded in trusted social relations and community logics, show how harm reduction becomes a process of collective world-building, where new norms, competencies, and care practices are co-produced, not merely delivered from above.

It is imperative to illuminate the specific risks that are unique to people who use IPEDs. As Alexis observed, while some commonly recognised concerns, such as needle sharing and hepatitis, may not be as prevalent within this demographic, there are distinct risks that do merit attention and provide the basis for informed harm reduction strategies.

Alexis [44, female]: They’re very different risks, there’s not the whole hepatitis. . . we’re not sharing needles. But there’s the other stuff of using vials that are too old, using needles in multiple vials and your infection control.

This highlights the pressing need to shift the focus from generalised intravenous safety practices to the nuances of intramuscular injection safety specific to people injecting IPEDs, which clearly differ and need to be addressed through continued research and understanding, to grasp the cultural language of these spaces.

Chicago [39, male]: So that’s probably the biggest hurdle, but learning what intramuscular injection is, it’s just injecting into the muscle and what sites you can go to. There’s even some sites you can only have a certain amount of mls you can put in, like certain amount of maybe like you’d put a larger volume into larger muscle groups essentially, but not a lot of people get told these things and they’re just like, well, I did that much in my leg or I did that much in my glute. I can do that in my delt or whatever.

STS scholarship (see Mol, 2002) reminds us that safety is not a universal standard but a relational practice, dependent on local norms, bodily routines, and the circulation of tacit knowledge. This is why conventional models of harm reduction often fail to map onto the lived, material realities of this group.

While discussions regarding people who inject drugs have tended to grapple with a well-documented set of risks primarily associated with shared equipment and blood-borne viruses, people who inject intramuscularly confront a different landscape of dangers, particularly those related to the handling and storage of their substances and equipment. Alexis underscored how storage habits, like leaving vials in unhygienic environments, contribute to the issues surrounding ‘safer injecting’, yet often go overlooked: *Alexis [44, female]: You’ve stuck this vial in your gym bag with your dirty socks and then shoved it in your mouldy bathroom cupboard.* The frequency of infections and the casual attitudes toward sterility highlight a major gap in ‘steroid literacy’ and safer practice. For instance, people injecting IPEDs may often overlook significant risks associated with cross-contamination and improper storage, which can be equally hazardous. Boaz emphasised the negligence that can arise from poor hygiene practices, noting: *Boaz [31, male]: Just basic shit. Like, don’t put it in your bag with your shoes. . . don’t leave it in a hot car for weeks on end.*

These remarks highlight ongoing knowledge gaps around safer intramuscular injection among both AAS consumers and service providers (Piatkowski et al., 2022, 2024a). Unlike intravenous use, harm reduction here must focus on sterility of substances and equipment. Tailored resources should build trust, embed lived experience, and use contextualised visuals to make risks and safer practices more relatable. Doing so recognises safer injecting as a situated, socio-technical practice, coming together not only through protocols but through bodies, relationships, and place-specific routines.

## Discussion

This analysis affords us new ways of knowing, understanding, and responding to the people who inject IPEDs. Our findings add weight to extant work, supporting the notion that safe injecting practices among this population are neither static nor solely determined by biomedical evidence; rather, they are dynamic, socially embedded, and shaped through embodied, everyday performances within IPED-using communities (Fomiatti et al., 2019; Underwood, 2017). By reframing ‘safer injecting’ as a sociotechnical *matter of concern* (Latour, 2004), we reinforce that these are not a set of *matters of fact*. That is, IPED injecting practices are assembled through attachments to devices (e.g., needles, vials, wipes), bodies, usage strategies and peer communities that circulate ‘how-to’ knowledge. In this framing, harm reduction emerges from what people care about and are attached to, rather than from decontextualised risk metrics. Such communal engagements foster a sense of shared responsibility and trust, which contrasts with traditional harm reduction services often characterised by top-down, clinical approaches (Fraser et al., 2020; McVeigh et al., 2016). Conceptually, these peer formations resemble the ‘publics’ that gather around contested topics, Latour’s (2009) notion of ‘Dingpolitik’, where techniques, bodies, and products become sites of deliberation and repair. This collective dimension underscores the current inadequacies and shortcomings associated with service design and delivery, demanding further attention and innovation.

The stigma surrounding injecting reinforced by cultural views of drug use, emerged as a recurring theme in interviews. Participants described injecting as a taboo subject, fostering misinformation and fear, even among experienced consumers. This stigma, particularly around infections and injection techniques, reflects broader societal perceptions of needles as ‘dirty’, aligning with Douglas’s (1966) concept of ‘matter out of place’. Extending Douglas via Latour (2004), we show how stigma adheres not simply to *dirt* but to assembled practices and objects (e.g., multi-use vials, visible injection-infection sites) that become *concernful things* around which authority and expertise are negotiated. These views hinder open discussions on safer injecting practices, as needles are seen as transgressive rather than tools for harm reduction. This finding is consistent with prior studies highlighting stigma as a significant barrier to effective harm reduction for IPED consumers (Cox et al., 2024; McVeigh et al., 2022), yet it also reveals nuances unique to this group, such as stigma internalised within peer networks and shaped by aesthetic and performance goals (Santos and Coomber, 2017). Concerns about infections, contaminated substances, and injection site reactions reflect the deeply embodied nature of these harms. Safer injecting practices are not only learned through formal education but are enacted and normalised through everyday exposure to information and the actions of those who inject IPEDs (Fraser et al., 2020; Seear et al., 2020). Much of the knowledge about safe injecting comes from informal networks like friends, partners, and coaches. In addition, more recently, social media platforms have provided a novel space for the sharing of such information (Cox and Piatkowski, 2024). While these informal networks provide critical support and a culturally relevant knowledge base (Gibbs et al., 2022; Piatkowski et al., 2023), they may also perpetuate misinformation or incomplete understanding, highlighting the challenge of integrating accurate, evidence-based information without undermining peer support systems (Havnes et al., 2019).

Our findings suggest that current harm reduction efforts, particularly those aimed at safer injecting, fail to adequately address the lived-living experiences of IPED-injecting communities. The persistent focus on sterility and infection management, while important, overlooks the broader social, cultural, and embodied dynamics influencing risky behaviours. This narrow focus ignores many of the elements that ‘matter’ to those who belong to this community, including health enhancement, body image, and performance goals (Fomiatti et al., 2019; Latham et al., 2019). Theoretically we, therefore, shift from delivering facts about risk to convening the concerns through which risk is made actionable, aligning Latour’s *matters of concern* with a care-centred orientation that asks what obligations and response-abilities are produced in practice (Piatkowski et al., 2024b). In doing so, we specify the mechanisms by which practices stabilise: (1) sociomaterial assemblages (devices, bodies, spaces) and (2) peer-communities that adjudicate credibility. These accounts contribute to theory by showing how safer injecting is an emergent property of these assemblages rather than an attribute of individual decision-making. Such gaps in service delivery contribute to the vulnerability of IPED consumers, as harm reduction approaches may appear irrelevant, inaccessible, or even counterproductive when they do not align with consumers’ lived realities and priorities (Havnes and Skogheim, 2019). We attempt to trace and excavate the components which might make safe injecting synonymous with ‘safer use’ and health enhancement here, thus providing new ways of responding to IPED injecting. Practically, this reframing implies design principles for services: assemble *with* consumers (co-design), enrol objects that materialise care (e.g., needle length and gauge considerations), and host moderated spaces where peers and clinicians can deliberate around shared concerns, an approach consistent with Latour’s (2009) call to *make things public*.

### Implications

Reframing our current ways of knowing involves recognising that much of the knowledge surrounding injecting IPEDs is already shared within communities, but we need to support and elevate the legitimacy of this knowledge, especially when it is focused on safer use and collective harm reduction. A more holistic understanding of safer use, one that aligns with the terminology and cultural language this group values, is essential. For people who use IPEDs, safe injecting is part of a broader practice of optimising the mechanism of delivery and drug distribution. Why does this matter? Because optimising these aspects directly improves the absorption and utilisation of substances, which in turn leads to better health outcomes – what we term *health enhancement*. While harm reduction often focuses on minimising risks (i.e., like infections and needle sharing), for people who use IPEDs, the conversation needs to shift toward optimising drug use to improve both physical and health outcomes. This is where we begin to delineate between the previous focus on harm reduction and, begin to trace what health enhancement can look like. These distinctions are critical as many in this community are driven by goals related to health and fitness (Santos and Coomber, 2017), believing these substances contribute to developing more muscle mass, reducing fat, and achieving aesthetic goals (Monaghan, 2001). We provide three key areas where concern could be re-configured, injection, needles, and aseptic techniques.

1. **Injection site selection** is crucial because it directly influences both the needle size and the volume of oil that can be safely administered. Each muscle has a different capacity to tolerate volumes of injected substances (Minto et al., 1997; Sartorius et al., 2010). For instance, the lateral deltoid, a smaller muscle group, may not accommodate more than 1 to 1.5 millilitres of oil in someone less experienced with IPED use (i.e., less muscular). Injecting more than that risks poor absorption, which could lead to tissue trauma, swelling, and infection (Banke et al., 2012; Schäfer et al., 2012). In contrast, larger muscle groups such as the vastus lateralis or gluteus maximus make them better sites for larger volumes (Cocoman and Murray, 2008). This careful attention to site selection is critical for ensuring proper absorption, which directly impacts drug efficacy and outcomes.
2. **Needle size and injection depth** are also pivotal. The correct needle must correspond to both the muscle group being targeted and the volume of oil being injected. Shallow injections, where the needle does not penetrate deeply enough into the muscle, can alter the drug’s release profile (Zuidema et al., 1988). For example, injecting androgens too shallowly can affect their half-life, which is designed to release the drug steadily over time (Kuhn, 2002; Kutscher et al., 2002). If the injection is too shallow or too deep, it may not distribute evenly within the muscle tissue, potentially causing hormone fluctuations. Uneven androgen levels can disrupt steady hormone balance, which in turn affects the rate of protein synthesis (Thiblin and Petersson, 2005) and, therefore, muscle gain. People often plan their injection schedules carefully to maintain hormonal stability, so errors in injection depth can compromise the entire cycle’s effectiveness.
3. **Aseptic techniques**, while sometimes perceived as secondary, these techniques are central to maintaining the integrity of the injection process. Failing to recognise that repeated puncturing of a vial can introduce contaminants from the environment or from the needle itself. Each time a vial is accessed, there is potential for pathogens to enter, particularly if aseptic techniques are not rigorously followed. Infections not only derail training progress but also lead to the formation of scar tissue, which can cause long-term complications (Rich et al., 1999). Bacterial infections may also require medical intervention, which could interrupt peoples’ carefully timed regimen, again affecting overall performance and goals.

By framing safe injecting as aligned with the community’s goals, we can legitimise their knowledge while offering targeted, peer-led education, through workshops, programs, or digital tools, to strengthen safety without undermining care networks.

### Limitations

This study provides important insights for improving harm reduction among people who use IPEDs but has several limitations. The sample was drawn from established community networks in Australia, which may limit generalisability to other locations with different services and cultures. Participants varied widely in their experience, from beginners to long-term consumers, and this diversity affects how harm reduction knowledge and practices are understood. The IPED community itself is highly heterogeneous, with participants using various substances such as anabolic steroids, peptides, and growth hormone. This variation influences risk profiles and harm reduction needs but is only partially captured here. Finally, while we describe the sample as broadly representative of the IPED injecting community, it likely underrepresents more isolated or marginalised individuals. These factors mean findings should be interpreted as reflecting a specific subgroup, highlighting the need for future research with broader, more diverse samples.

### Conclusion

Our findings challenge the notion that safe injecting practices for people who use IPEDs, particularly AAS, can be addressed through purely biomedical or instructional approaches. Injecting practices are shaped by social networks, embodied experiences, and cultural taboos; thus, necessitating a community-driven harm reduction strategy. Therefore, we advocate for interventions that are sensitive to the needs of the IPED community. Future harm reduction initiatives must go beyond simply providing information or sterile supplies; they must also foster spaces where the stigma surrounding injecting can be challenged, and where accurate, community-relevant knowledge can be shared and practiced. Importantly, as Latour’s notion of matters-of-concern suggests, the social and political dimensions of these interventions must be acknowledged, ensuring that harm reduction strategies are responsive to the evolving needs and dynamic nature of the community. This redefinition of safer injecting practices opens pathways for more effective, equitable, and sustainable harm reduction strategies in the future (Table 1).

**Table 1.** Participant information.

Participant	Pseudonym	Age	Gender
1	Drake	29	Man
2	Raymond	42	Man
3	April	28	Woman
4	Anwir	37	Man
5	Alexis	44	Woman
6	Petir	40	Man
7	Hoyt	41	Man
8	Gavin	32	Man
9	Boaz	31	Man
10	Oscar	39	Man
11	Ciaran	32	Man
12	Jasmine	44	Woman
13	Chicago	39	Man
14	Damon	27	Man
15	Xander	28	Man
16	Eben	37	Man
17	Rosa	45	Woman
18	Cherry	33	Woman
19	Dan	34	Man
20	Bruce	31	Man
21	Alder	40	Man
22	Hugo	49	Man
23	Sean	22	Man
24	Niel	28	Man
25	Charlie	41	Man
26	Ed	42	Man
27	Ross	32	Man
28	Juniper	52	Woman
29	Lilith	44	Woman
30	Lena	44	Woman
